# Research on blasting mechanism and blasting effect of aqueous media in open pit coal mines

**DOI:** 10.1038/s41598-023-46449-6

**Published:** 2023-11-06

**Authors:** Xiaohua Ding, Xin Liu, Zhongchen Ao, Hao Qin, Xiaoshuang Li, Kexin Huang, Shuangshuang Xiao, Mao Wu, Donghua Zhang, Chun Zhu

**Affiliations:** 1 School of Mines, China University of Mining and Technology, Xuzhou, 221116 China; 2 School of Civil Engineering, Shaoxing University of Arts and Sciences, Shaoxing, 312010 China; 3 Zhejiang Transportation Resources Investment Group Limited Mining Branch, Hangzhou, 310000 China; 4 School of Energy, Xi’an University of Science and Technology, Xi’an, 710064 China; 5 China Coal Pingshuo Group Co., Ltd., Shuozhou, 036006 China; 6 School of Mining Engineering, Taiyuan University of Technology, Taiyuan, 030024 China; 7 School of Earth Sciences and Engineering, Hohai University, Nanjing, 210098 China

**Keywords:** Environmental impact, Environmental sciences, Materials science

## Abstract

Surface coal mining procedures include piercing—blasting—mining and loading—transportation—discharging, blasting link exists due to the poor blasting effect leads to low loading efficiency, blasting dust caused by environmental pollution and other problems. In this paper, from the mechanical characteristics of the water medium, we analyze in detail the transferring effect, transducing effect and bubble pulsation phenomenon of the water medium in the blasting process. The results show that when the blasting medium is water medium, the maximum principal stress is 1.53 times that of air medium; the peak energy transfer can be up to 2.73 times that of air medium. With the help of TrueGrid/LS-DYNA finite element analysis software to simulate the dynamic process of blasting, the study of the maximum principal stresses around the hole, the top of the slope, the foot of the slope on the maximum principal stress changes, the results show that the maximum principal stresses around the hole, the top of the slope, the foot of the slope unit with the increase in the water content is gradually increasing trend. Finally, combined with the actual mine production conditions for blasting field test, water-mediated blasting dust reduction rate of 75%, the use of AHP—fuzzy comprehensive evaluation method of two groups of traditional dry hole blasting and three groups of water-mediated blasting comprehensive evaluation, the results show that the water-mediated blasting scores are higher than the traditional dry hole blasting, proving that the water-mediated blasting has a certain prospect of engineering applications.

## Introduction

Opencast mining has the advantages of high resource recovery rate, good safety conditions, high labour efficiency and large production scale. When producing the same capacity of coal, the cost of opencast mining is mostly lower than other methods, which meets the needs of China's current energy transition stage. In order to achieve energy conservation and efficiency and to promote the country's energy transition, there is a need to develop and research on opencast step blasting and apply advanced technologies to obtain greater economic and social benefits, etc. The existing step blasting method is single and the blasting effect is not ideal, so this paper proposes aqueous media blasting, using water as the medium to spread the blast pressure to destroy the vessel, which can produce a reflection effect to form a secondary loading, intensifying the destruction of the vessel wall, thus making the vessel evenly disintegrated and broken. At the same time, aqueous media blasting by the special characteristics of the internal presence of aqueous media, can improve the utilization of blasting energy in the explosion while reducing the blast generated by carbon dioxide, harmful gases, blasting dust, etc., has practical engineering significance.

Research scholars have accumulated a large number of results in the direction of aqueous media blasting, among which Yang^1^. The technique of confined blasting in water-filled deep holes is proposed as a measure to prevent high rock pressure. Wang, et al.^2–4^. The dynamic process of dust generation during blasting was recorded using a high-speed camera, and a dust reduction method based on water-sealed blasting was proposed by using a gray-scale recognition method in MATLAB to recognize the pictures. Huang, et al.^5^. The use of hydraulic blasting combined with hydraulic fracturing proved to be superior to conventional pre-fracture blasting. Li, et al.^6^. The blasting disturbance of different coupling media was compared, and the results show that the disturbance effect of water-coupled blasting is 1.32 times that of air-coupled blasting. Yang, et al.^7^. A pressurized water-coupled blasting technique is proposed, which realizes the combination of ultra-dynamic explosive load and static load effect of pressurized water. Fan, et al.^8^. The results of the coupled loading under different water depth conditions were calculated showing that the extent of the crushed and fractured zones under shallow water conditions is slightly larger than that of the crushed and fractured zones under no water conditions. Cui^9^, a new method using water-silt composite blasting is presented, which improves the rock fragmentation rate, reduces rock buildup, lowers dust, and saves explosives. Jia et al.^10^ carried out numerical simulation of blasting on coke rock steps using (LS-DYNA) and concluded that the root cause of the high dust production from blasting operations is the excessive fragmentation of coke rock after the blast wave action. Song, et al.^11, 12^ conducted dynamic compression tests on lignite with different impact velocities, and the results showed that the increase of strain rate had a significant hardening effect on the dynamic mechanical parameters of the rock samples. Zhang, et al.^13, 14^. The finite element program LS-DYNA was used to model the double millisecond holes of tabletop blasting, which provides a basic theory for the protection of buildings near the blasting source. Wang et al.^15–17^ used LS-DYNA software to establish the numerical model of different hole diameters and hole spacing and verified it in the field. After blasting, the slope was smooth and level, and the semi-perforation rate was guaranteed to be more than 90%. Yin et al.^18^ used ANSYS/LS-DYNA simulation to study the stress distribution of 24 m high platforms with different spacing parameters.

Scholars at home and abroad have conducted in-depth studies on the dust initiation mechanism and dispersion mechanism of blasting dust. Among them, Li, et al.^19^ used AERMOD software to simulate dust diffusion, establish dust concentration evaluation and diffusion modeling, and obtained contour plots of dust concentration around coal mines. Adushkin et al.^20, 21^ presented gas dynamics calculations of dust and gas cloud elevations after blasting, pointing out the effect of large-scale blasting on regional seismic activity. Khazins^22, 23^, studied the initial stages of the formation of thermally dilute dust and gas clouds after the scattering of the explosion products, obtaining a relationship between the height of ascent and the size of the gas and dust clouds and the distance between neighboring explosions. Yin, et al.^24^ developed a pollution evaluation system with a gray scale averaging algorithm (G(a)) in digital image processing technique to determine the existence of three fitted correlations between the parameters of soot and diffusion time. Huang, et al.^25^ studied the mechanism and migration law of blast dust removal, and the results showed that in the stage of impact movement and mushroom cloud formation, the explosive gas generated by the blast expands rapidly, and a large amount of dust is rushed into the atmospheric space under the kinetic energy of the blast impact. Wang, et al.^26^ presented a highly reliable model about the influencing factors and diffusion model of blasting dust diffusion.

Many factors influence the evaluation of the blasting effect, and many scholars have conducted in-depth research on this issue based on various models and methods. Yang, et al.^27, 28^ optimized the blasting parameters, and several sets of blasting tests were carried out in the field, which greatly improved the large block rate and average block satisfaction rate, and verified the feasibility of the optimized scheme Fu, et al.^29^ developed a rock blasting crushing control model by analogous theory in the laboratory. Babaeian et al.^30, 31^ proposed a method for evaluating and predicting fragmentation based on image analysis methods. Bamford, et al.^32^ investigated the application of Unmanned Aerial Vehicle (UAV) systems in monitoring and improving the blasting process in open pit mines.

Aqueous media blasting is a potential research direction to improve the blasting effect of open pit mine rock, as seen in other areas of aqueous media blasting research. However, some research has been carried out on hydro blasting in open pit mines. Therefore, it is necessary to carry out in-depth research on the mechanism of deep hole aqueous media blasting to achieve the optimisation of the blasting effect on the mine rock. This paper investigates the mechanism and application of aqueous media blasting, using LS-DYNA software to simulate the entire blast process variation, not only to enrich the theoretical knowledge of step blasting in open pit mines, but also to provide new options for blast design. The simulations are used to optimise blasting methods in opencast coal mines to improve blasting effectiveness, reduce explosive unit consumption and achieve energy savings and efficiency gains. The special characteristics of aqueous blasting with the presence of aqueous media inside the blast hole are of practical engineering importance as it increases the blast energy utilisation and produces a water mist that reduces the amount of dust, carbon dioxide, and harmful gases generated by the blast.

## Materials and methods

This paper investigates the mechanism of the effect of aqueous media on the dust generated by blasting and the blasting effect, simulates a charge structure with good dust reduction and blasting effect for field tests, and analyses the economic and technical impact of aqueous media blasting on step blasting. The study was completed using a combination of rock explosion dynamics, rock mechanics and theoretical foundations of step blasting in opencast mines, using numerical simulation methods to guide optimisation and the design of relevant indoor and field tests to complete the study of aqueous media blasting.

### Numerical simulation of the transfer action of aqueous media

**Figure 1:**
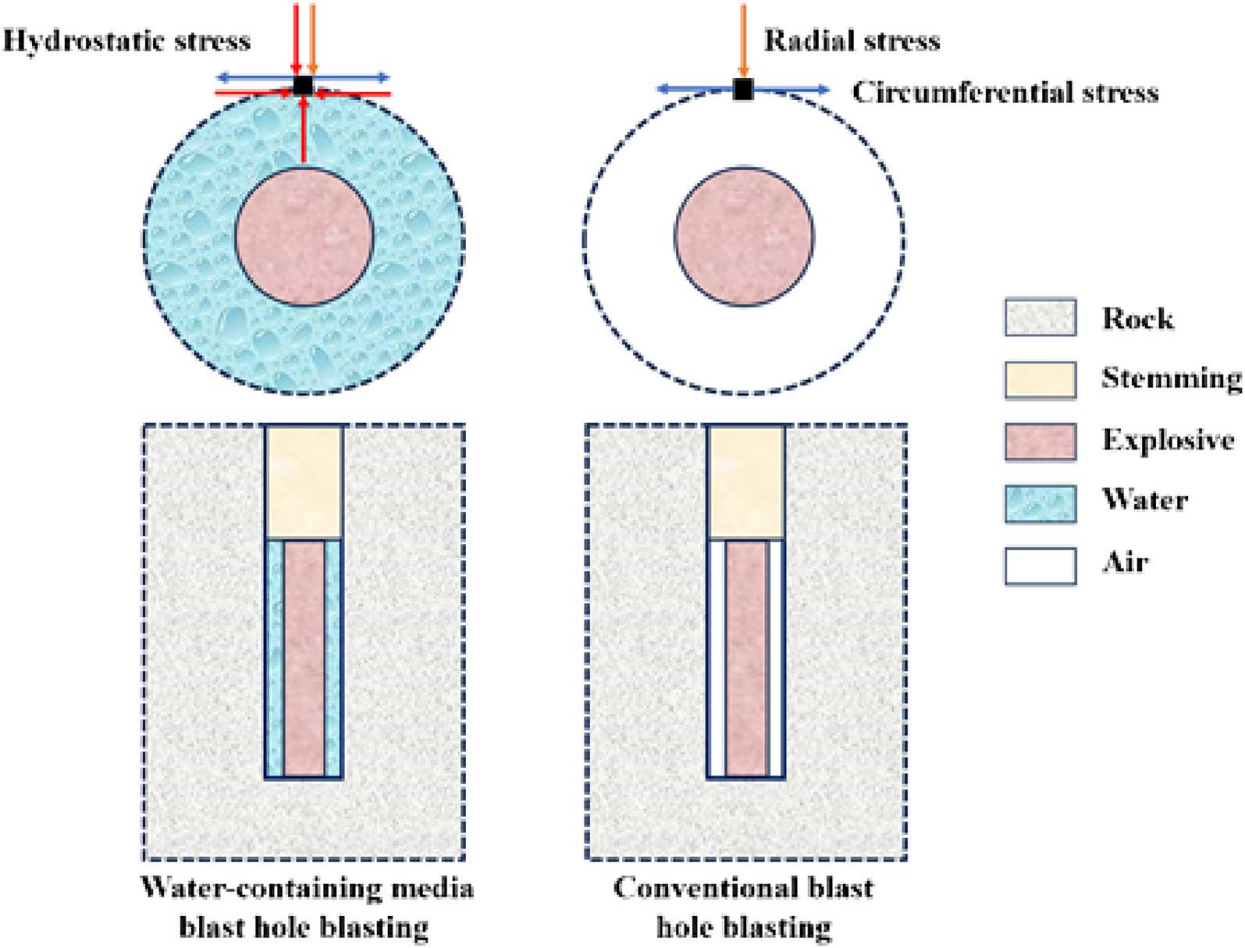
Water-mediated blasting and conventional blasting hole blasting mechanism.

Water-mediated blasting refers to the gap between the explosive and the hole wall filled with water blasting method, water-mediated blasting and traditional dry hole blasting mechanism is different from that shown in Fig. 1. Has the advantages of improving the effective utilization of explosive energy, reduce blasting hazards, reduce construction costs. Due to the role of hydrostatic pressure, blasting rock in the process of moving to overcome the hydrostatic pressure and the medium resistance of water, in the propagation of stress waves, compared with the rock-air interface, in the rock-water interface caused by the reflection of the tensile stress is much lower. At the same time, due to the high density of water, flow viscosity, blasting stress waves in the water attenuation rate is slower than in the air, water-mediated blasting transferred to the rock explosion pressure and explosion energy is greater. In addition, the compressibility of water is much lower than air, so in the case of the same charge, the blasting stress wave stimulated by water is higher.

**Figure 2:**
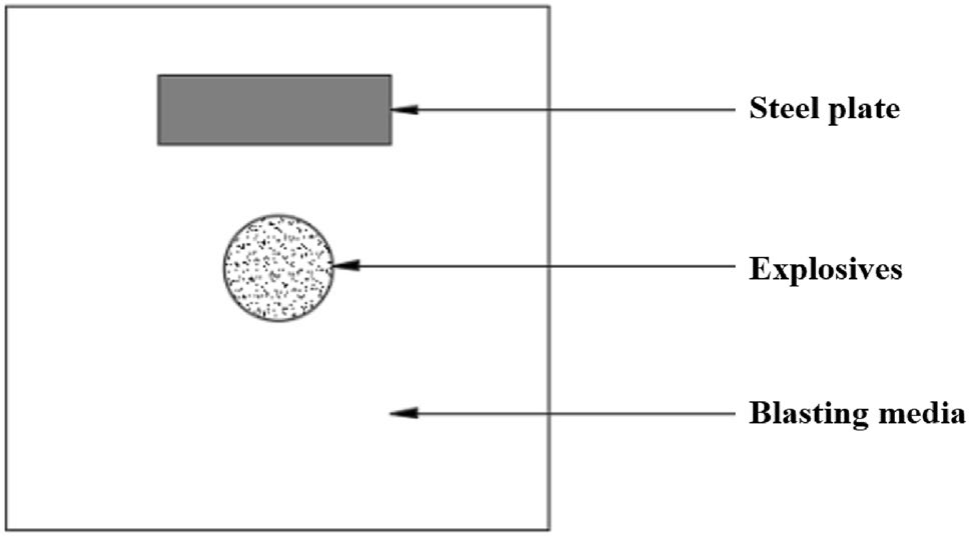
Calculation model structure of blasting media action.

A numerical model of the explosion is established, with the centre of the model being the explosive sphere and a solid steel plate set 1.5 m from the centre to map the transfer of the blasting medium as shown in Fig. 2.

In the modelling, the materials were coupled using the fluid–solid coupling algorithm, the blasting media boundary was set as a non-reflective boundary, the explosive material was *MAT_008, the air and aqueous media was *MAT_009 and the steel plate was *MAT_003, and the appropriate equation of state was used in the calculation process. The explosive material parameters are shown in Table 1.

**Table 1:** *MAT_008 TNT material parameters table.

Parameters	Meaning	Value	Parameters	Meaning	Value
MID	Material identification code	1	RO	Mass density	1630.0
D	Explosive speed	6930.0	PCJ	Explosive pressure	2.1e^+12^
BETA	Explosive burning option	0.0	K	Volumetric modulus of elasticity	0.0
G	Shear modulus	0.0	SIGY	Yield stress	0.0

For a water medium, choose *EOS_GRUNEISEN, which defines the pressure of the compressed material in terms of the Grueneisen equation of state. For air, the equation of state is *EOS_LINEAR_POLYNOMIAL, which is linear in internal energy. The material parameters for water and air media are shown in Table 2.

**Table 2:** *MAT_NULL Table of material parameters for aqueous and air media.

Parameters	Meaning	Value	
		Aqueous media	Air media
MID	Material identification code	2	3
RO	Mass density	998.21	1.2929
PC	Pressure cut-off (≤ 0.0)	− 10.0	0.0
MU	Dynamic viscosity μ	8.684e^−04^	0.0
TEROD	Relative volume. v/v0, for erosion in the stretched state	0.0	0.0
CEROD	Relative volume. v/V0, for erosion in the compressed state	0.0	0.0
YM	Young's modulus (for hollow beam and shell units only)	0.0	0.0
PR	Poisson's ratio (for empty beam and shell units only)	0.0	0.0

The steel plate material parameters are shown in Table 3.

**Table 3:** *MAT_003 sheet material parameters.

Parameters	Meaning	Value	Parameters	Meaning	Value
MID	Material identification code	3	RO	Mass density	7800.0
E	Modulus of elasticity	2.1e^+11^	PR	Poisson's ratio	0.3
SIGY	Yield strength	4e^+8^	ETAN	Shear modulus	1e^+9^
BETA	Hardening parameters	1.0	SRC	Strain rate parameter C	0.0
SRP	Strain rate parameter P	0.0	FS	Effective plastic strain	0.0
VP	Rate effect formula	0.0			

### Explosive bubble pulsation phenomenon in water

Explosives detonated in the water instantly transformed into a rapidly expanding high-temperature, high-pressure air mass, and the formation of shock waves propagating outward in the water, a period of time due to inertial expansion of the bubble, when the bubble expands to the maximum time, the pressure inside the bubble is less than the surrounding hydrostatic pressure to form a negative pressure overpressure, the periphery of the water back to the extrusion of bubbles until compressed to the smallest diameter, which, again, will be found inside the bubble pressure is higher than the external pressure, and so the bubble will start a new round of expansion and contraction cycle. So that the bubble and start a new round of expansion and contraction of the cycle process. So repeated many times to form the bubble pulsation phenomenon.

Underwater blast loads undergo multiple stages of change, and due to the special mechanical properties of the aqueous medium, the number of bubble pulsations can be as many as ten or more, far exceeding the number of pulsations of an explosion in an air medium. This paper will demonstrate the multiple pulsations bubbles phenomena in underwater explosions using numerical simulations.

**Figure 3:**
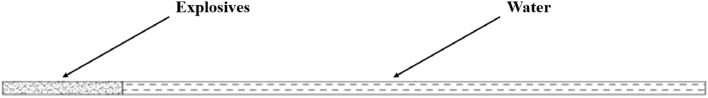
Structure of the numerical simulation of bubble pulsation.

A numerical simulation model of an explosive blast underwater is established, assuming equal pressure in all directions around the explosive, which simplifies the model to a spherically symmetric model, and therefore a one-dimensional spherically symmetric model is established, as shown in Fig. 3. In order to demonstrate the bubble pulsation of underwater blasting, the blast point is set at a certain distance from the water surface, so that the depth of the blast point is much greater than the maximum bubble radius, and the difference between the hydrostatic pressure above and below the bubble is small, and gravity can be ignored in the bubble migration analysis. The viscosity coefficient and other related parameters are set by default, the explosive explosion problem involves large deformation, so the explosive and water are set as ALE units, using the multi-material Euler algorithm. The material models for the explosive and aqueous media refer to the material types applied in Tables 1 and 3 of this paper.

### Numerical simulation model design for blasting

#### Design for different water contents in natural aqueous media pores

**Figure 4:**
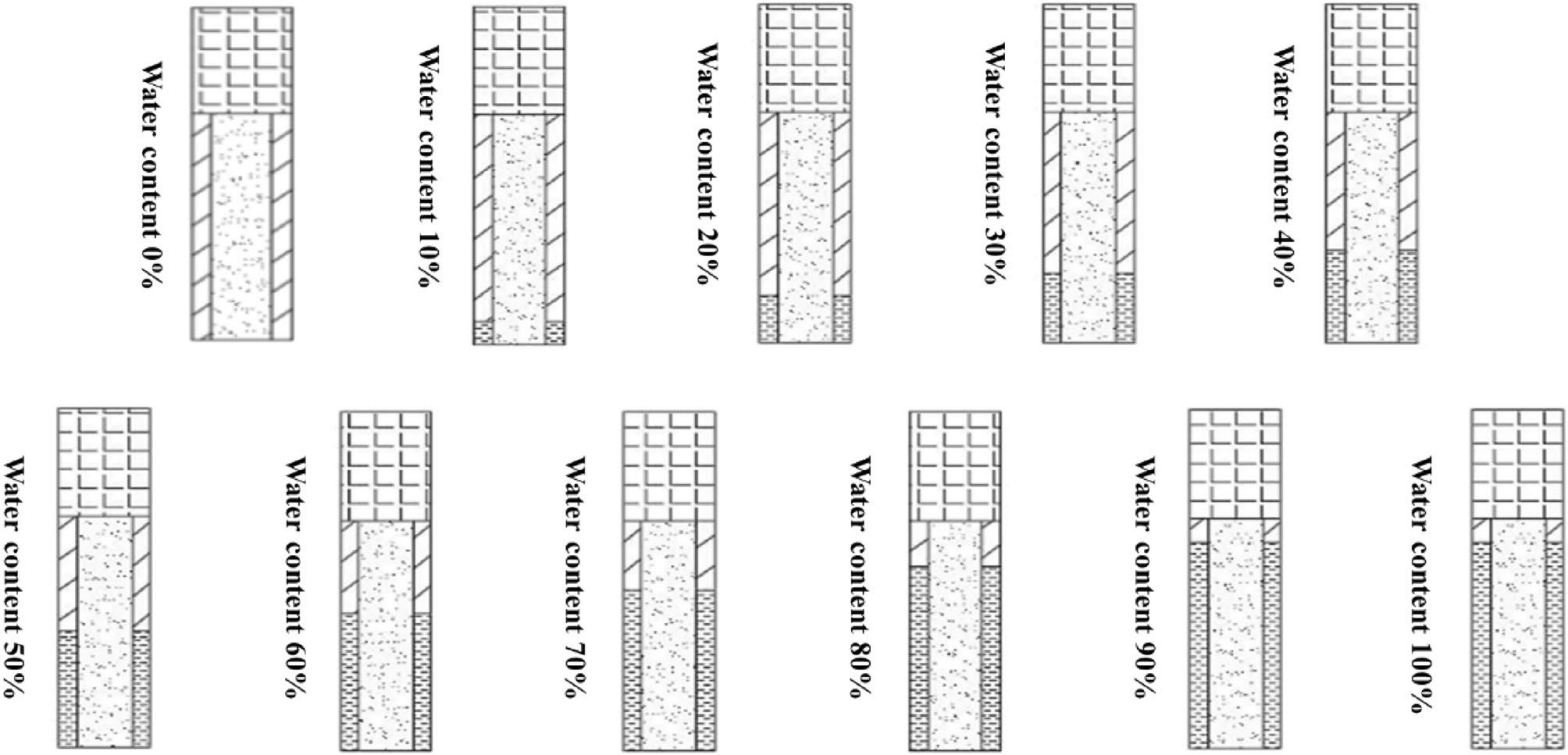
Structure of natural aqueous media borehole shell hole.

In an aqueous media hole, the natural presence of aqueous media will change over time, so the variable is the amount of water inside the hole. This will produce a different blasting effect, so the main variable in the charging structure of a water-laden hole is the water content inside the hole. After determining the explosive charge and blockage length, the remaining space is the free volume of uncoupled charge and the water content percentage is the amount of free volume occupied by the aqueous media. The detailed gun hole structure is shown in Fig. 4.

#### Design of geometric parameters for numerical simulation of blasting

Simulations were carried out using the number TrueGrid/LS-DYNA software to investigate the different distributions of the aqueous media inside the gun bore and the different charge structures.

The model parameters were set as follows:

(a) the borehole diameter (d) was 250 mm; the step height (H) was 15 m; and the step slope angle (α) was 70°;

The drilling method is vertical, which is often used in large mines; the layout of the holes is staggered in a triangular pattern.

Over-deepness of the shell hole:

1 $$h = (5 \sim 10)d = 1.25\,\,{\text{m}} \sim 2.5{\text{m}}$$

where h is the depth of gunhole, m;

Chassis resistance line:

2 $$W_{1} = (0.6 \sim 0.9)H = 9\,{\text{m}} \sim 13.5\,\,{\text{m}}$$

where W1 is sump resistance line, m;

Filling length:

3 $$l_{2} = (0.7 \sim 0.8)W_{1} = 6.3 \sim 9.45\,\,{\text{m}}$$

where l2—length of fill, m;

The distance from the centre of the borehole to the top line of the slope is generally B ≥ 2.5 ~ 3.0 m, which is taken as 4 m in the model.

Considering the actual situation, a multi-borehole model is needed to calculate the parameters of the hole network as follows:

Hole spacing:

4 $$a = mW_{1} = 10.8 \sim 20.25\,\,{\text{m}}$$

where a—hole spacing, m; m—coefficient of the density of the shell hole, generally taken as m = 1.2–1.5;

Row spacing b:

5 $$b = a \cdot \sin \,\,60^{ \circ } = 9.35 \sim 17.54\,\,{\text{m}}$$

where b—row distance, m.

Therefore, in the model design of this paper, the uncoupling factor is chosen to be 1.25, i.e. the charge diameter d2 is 200 mm when the charge is not coupled. Further values are taken for the model parameters, as detailed in Table 4.

**Table 4:** Table of geometric parameters of the blast model.

Parameter	Value	Parameter	Value
Step height (*H*/m)	15	Step slope angle (*α*/°)	70
Diameter of the cannon hole (*d*_1_/mm)	250	Charge diameter (*d*_2_/mm)	200
Distance from the centre of the borehole to the top line of the slope (*B*/m)	4	Pattern of holes	Triangular fabric holes
Chassis resistance line (*W*_1_/m)	10	Gun hole length (*l*_1_/m)	17
Filling length (*l*_2_/m)	7	Extra deep gun holes (*h*/m)	2
Hole spacing (*a*/m)	12	Distance between rows (*b*/m)	10

**Figure 5:**
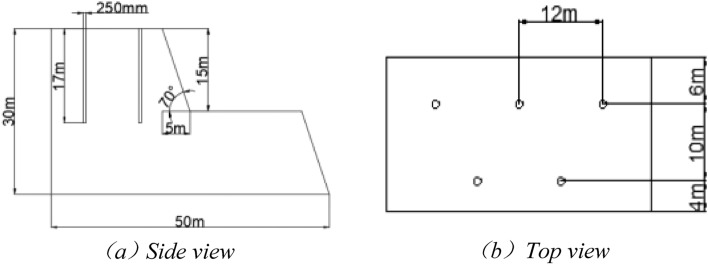
Parameter diagram of the hole network.

Details of the gun hole layout are shown in Fig. 5.

**Figure 6:**
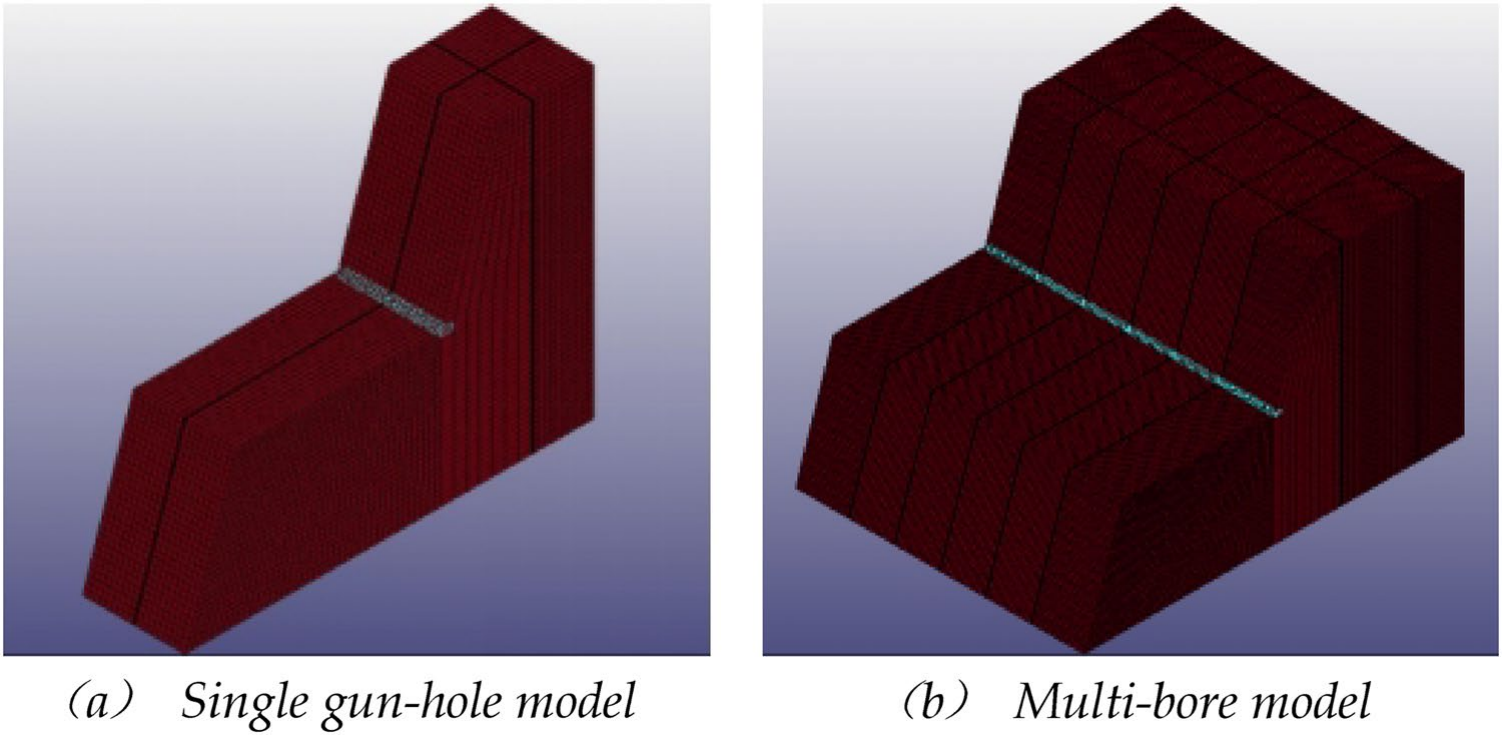
Schematic diagram of the foot of the blast step unit.

**Figure 7:**
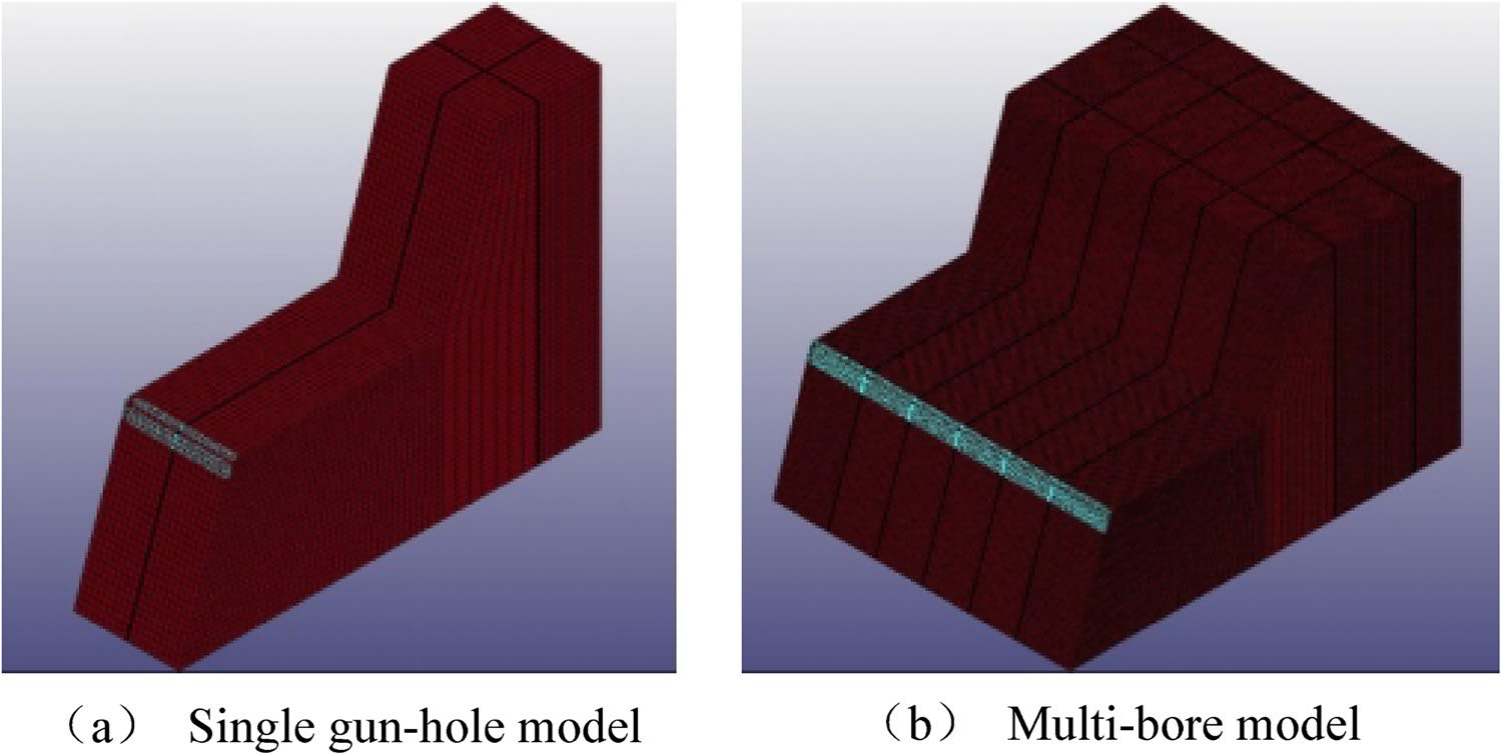
Diagram of the top unit of the next step of the blast step.

The actual blasting on site occurs across the entire slope, with multiple steps present. The shock wave generated by each blast not only loosens and breaks the blast step, but also affects its adjacent steps, with blast-generated stresses acting on the whole slope. In this paper, the unit at the foot of the blast step and the unit at the top of the next step of the blast step will be selected as the object of study, and the detailed locations are shown in Figs. 6 and 7. The maximum principal stresses in this unit are extracted as indicators for analysis.

During the modelling process, the grid is uniformly distributed at a reasonable density, and the locations of large strains in the blasting process, such as the location of the blast hole and the blasting media, are encrypted to ensure that the calculation process is close to reality and to reduce errors while improving the efficiency of the calculation.

#### Design of material parameters for numerical blast simulation

After establishing the geometric model using the TrueGrid software, the various materials were assigned parameters and boundary conditions were set by writing K files, and the model was calculated in Kg-m-s.

(1) Rock material parameters.

The overlying rock layers in the area where the open pit mine is located are mainly gneiss, quartzite and amphibolite, and their rock mechanics parameters are shown in Table 5. In order to obtain a general conclusion, the mean values of the strength of the above three rock types will be used as parameters for the numerical simulations throughout the theoretical study. The *MAT_003 (*MAT_PLASTIC_KINEMATIC) model was chosen for the operation and the detailed assignment parameters are shown in Table 5:

**Table 5:** *MAT_003 Rock material parameters table.

Parameters	Meaning	Value	Parameters	Meaning	Value
MID	Material identification code	1	RO	Mass density	3030.0
E	Modulus of elasticity	8.07e^+10^	PR	Poisson's ratio	0.23
SIGY	Yield strength	2.613e^+8^	ETAN	Shear modulus	1.760e^+7^
BETA	Hardening parameters	1.0	SRC	Strain rate parameter C	0.0
SRP	Strain rate parameter P	0.0	FS	Effective plastic strain	0.05
VP	Rate effect formula	0.0			

(2) Aqueous and air media

The material models and equations of state for the aqueous and air media in this section refer to Tables 1 and 2 in this paper.

(3) Explosives

The explosives selected were watertight emulsified explosives, taking into account that the blasting of aqueous media was to be carried out. Material type 8*MAT_008 (*MAT_HIGH_EXPLOSIVE_BURN) was chosen as the material model for the emulsion explosive as shown Table 6:

**Table 6:** *MAT_008 Table of material parameters for emulsified explosives.

Parameters	Meaning	Value	Parameters	Meaning	Value
MID	Material identification code	4	RO	Mass density	1300.0
D	Explosive speed	4000.0	PCJ	Explosive pressure	9.9e^+9^
BETA	Explosive burning option	0.0	K	Volumetric modulus of elasticity	0.0
G	Shear modulus	0.0	SIGY	Yield stress	0.0

### Field blasting tests for blasting in aqueous media

After completing a detailed study of the charging scheme, blasting tests were carried out in the minefield in the Pinshuo mining area of Shanxi Province. The test blasting step chosen was a rock stripping step, mainly sandstone with fine sandstone, medium sandstone and siltstone, where rock samples were taken for rock strength testing and detailed rock parameters were measured as shown in Table 7:

**Table 7:** Mechanical parameters of test mine rocks.

Rock name	Compressive strength (MPa)	Tensile strength (MPa)	Cutting strength (MPa)	Modulus of elasticity (GPa)	Density (kg/m^3^)
Sandy mudstone	16.8	0.95	3.62	8.3	2492
Fine sandstone	23.7	1.52	4.22	19.0	2521
Medium sandstone	28.9	1.37	3.21	15.2	2468
Siltstone	30.7	1.36	3.56	15.4	2575

The holes are drilled in a rectangular pattern with a vertical drilling direction, using high precision non-electric detonators for inter-hole delay and inter-row delay timing for surface blasting. Once the continuous charge is completed, rock chips from the borehole will be used as plugging material in situ.

#### Test scheme design

According to the field perforated blasting construction technology, the charging structure of the dry hole was selected, and the water medium was added to the hole by filling the water bag. According to the formula for calculating the optimal value of the mass ratio of water medium and explosives, the optimal loading capacity of water medium in the test bench was calculated to be 1.7 m.

The materials to be prepared are shown in Table 8.

**Table 8:** Aqueous media blasting test tools.

Tool name	Uses
Aqueous media blasting water bags	Vessel for aqueous media in the gun bore
Regular woven bags, bubble wrap, tape	Safeguarding water bags from damage by aqueous media transducer blasting
Bucket or container	For filling with aqueous media
Rope	Used to slowly lower the water bag into the shell hole to reduce the impact
Air spacers	Provide support between the aqueous medium and the explosive to secure loading
Industrial salt	Add to water in cold weather to prevent the aqueous medium from freezing

(1) Blasting water bag filling preparation.

**Figure 8:**
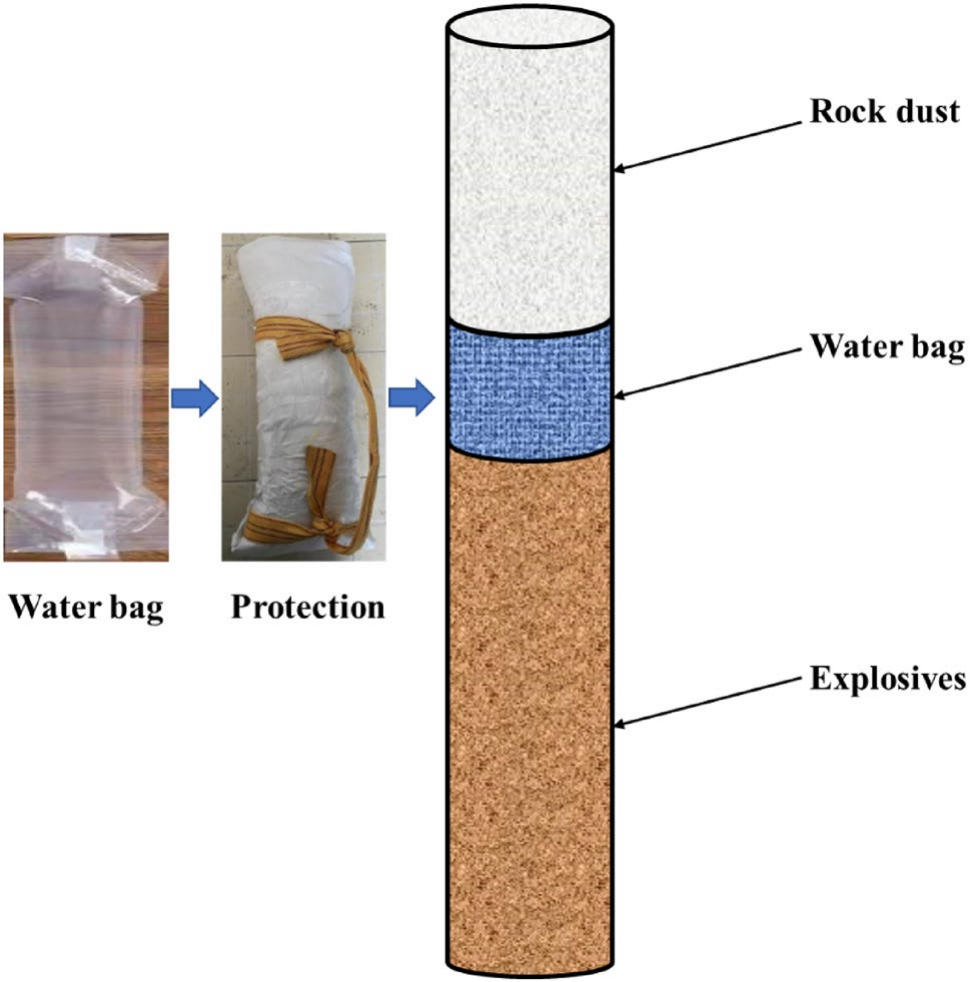
Structure of aqueous media blasting water bag.

Select biodegradable plastics as raw materials, can be naturally degraded after disposal will not cause pollution to the environment, length, width, thickness were 75 cm, 25 cm, 230 μm water bag, as shown in Fig. 8, to avoid the water bag in the handling of the loading was scratched, in the external increase in the protection measures, the choice of the water bag in the external increase of a layer of biodegradable anti-collision foam paper and biodegradable bio-woven bags, the water bag loading method using the water bag in the external winding rope knots, when loading with a rope hanging slowly into the shell hole. The water bag is filled by wrapping a knot around the outside of the water bag, and then slowly put into the shell hole with a rope when loading. Before the actual implementation, a trial loading was carried out in the cannon area to verify that the protective measures had no influence on the loading and the water bag could be placed smoothly. Choose the loading structure for the water bag is located in the explosives above the hole 1/3, the detailed structure shown in Fig. 8:

### Comprehensive effect of blasting of shell holes in aqueous media

#### Water-containing media blasting dust reduction effect

This field test will use a high-speed camera as in Fig. 8 to take pictures frame by frame, and analyse the dust diffusion and transport during blasting based on the images. MATLAB software will be used to count the distribution of the selected blasting pictures in terms of the distribution of grey values, and the different grey values will be used to represent the size of dust concentration, and the range of distribution of grey values in the pictures will be counted for concentration change characteristics analysis. Since the dust grey value has a threshold intersection with other non-dust areas, the dust distribution result of the first un-blasted picture was selected as the initial dust concentration reference standard, and the corresponding error correction was made for each picture after blasting.

#### Evaluation of the effect of water-containing media blast hole blasting

**Figure 9:**
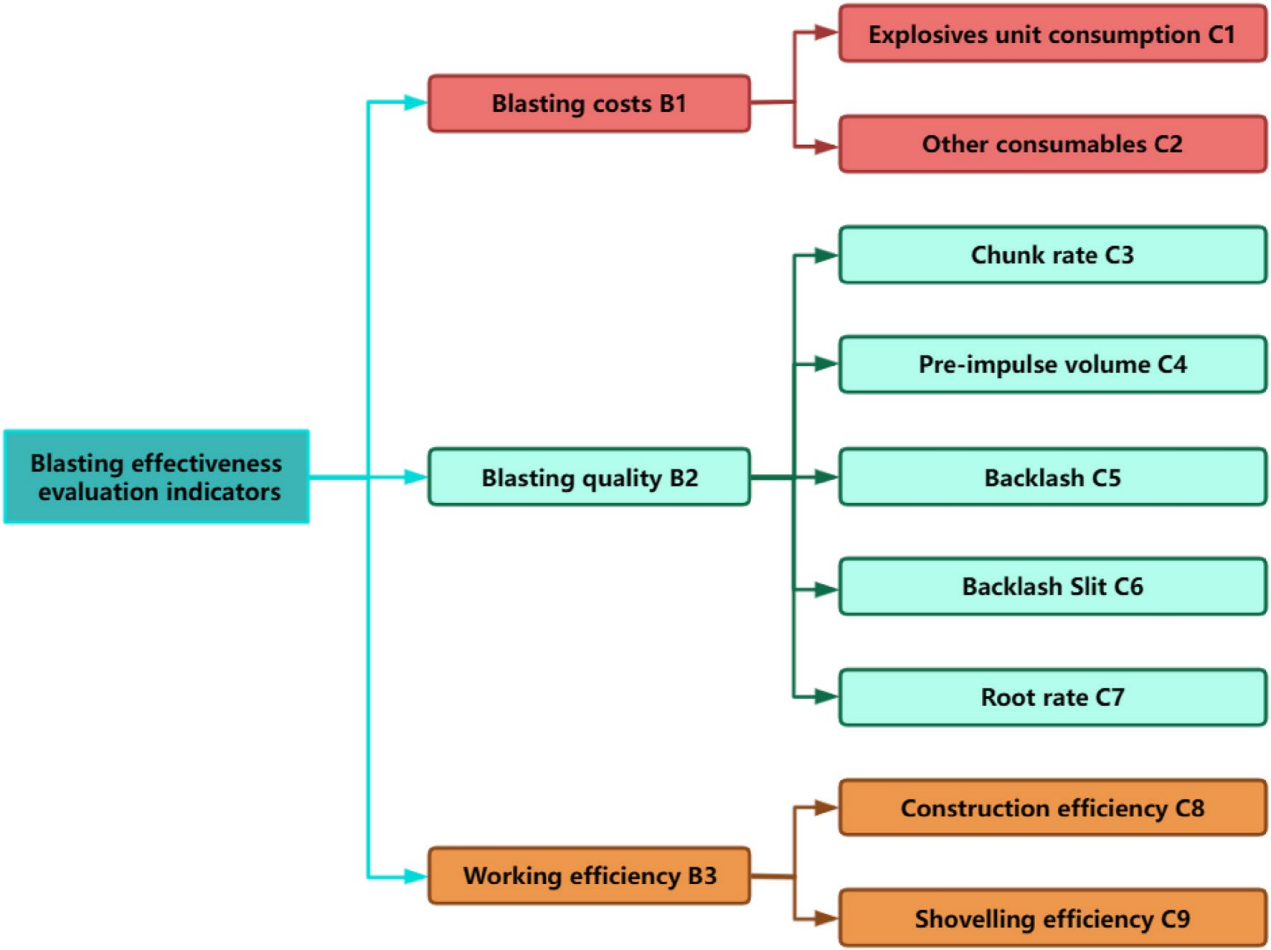
Hierarchy of blasting effect evaluation indicators.

After the completion of the field test and results of measurement, first through the survey data to obtain the three mines are most concerned about is also the most representative of the blasting effect of the indicators as a blasting effect evaluation of the first level of indicators: blasting quality, blasting costs and efficiency, and then the indicators continue to be divided into the rate of large blocks, the amount of front punch, the amount of backlash, the backlash fissure, the root rate, the explosives consumed, the other consumables, the construction efficiency, as well as shovel efficiency of the nine second-level indicators. After that, the evaluation matrix of single-indicator measurement with rationality is established, and the weights of each indicator are determined directly by entropy weighting method. Finally, the AHP-fuzzy comprehensive evaluation model was established to comprehensively evaluate the effect of blasting on the whole water-containing medium, and the structure of the AHP-fuzzy comprehensive evaluation model is shown in Fig. 9.

(1) Bulk rate: the proportion of the blast volume that is completed with a rock block size exceeding the maximum size classified for the blast job. In this paper, Split-desktop digital image analysis software is used to analyse the blockiness of the blast pile. It is also evaluated in conjunction with the calculation formula, namely:

6 $$\varphi \le 0.8\sqrt[3]{V}$$

7 $$\varphi \le 0.8A$$

where ψ—the maximum block size of ore rock allowed, m; V—bucket capacity of the excavator, m^3^; A—minimum size of crusher entrance, m.

(2) Forward impulse: after the blasting work is completed, the blast pile from the blasting area boundary towards the minimum resistance direction of the distance, this paper uses mine measurement equipment to obtain coordinates and then processed to obtain the forward impulse distance.

(3) after the amount of impulse: the blasting caused by the rock body to the minimum resistance direction of the opposite direction of the distance, the measurement method, and the same before the amount of impulse.

(4) Backwash fissures: rock fissures created on the slope of the unexploded steps on the back side, which are scored according to the evaluation of field technicians.

(5) Root rate: protruding above a certain height of the hard can and rock ridge of the extraction workings, known as the root, this paper uses the formula calculation to quantify the root, namely:

8 $$\delta_{G} = \frac{{\Delta h_{i} - h_{S} }}{{h_{S} }} \times 100\%$$

where δG—root rate; Δhi-average step level height, m; hS—standard step height, m.

(6) Explosives consumption: the consumption of explosives per unit square quantity of blasting work, calculated based on the blasting square quantity and the amount of explosives consumed.

(7) Other consumables: the cost of blasting equipment such as detonators, detonating cord, and other blasting consumables, obtained from site statistics.

(8) Construction efficiency: After the drilling work is completed, the holes need to be filled and stuffed and are evaluated on the basis of the time taken during the filling of the holes at the site.

(9) Shovel loading efficiency: The blasting effect directly affects the rate of shovel loading, which in turn affects the later transport and propulsion work, and is evaluated by the shovel loading capacity of the blast pile at the site.

### Informed consent statement

Informed consent was obtained from all subjects involved in the study.

## Results and discussion

### Numerical simulation results of the action of the water medium transfer

**Figure 10:**
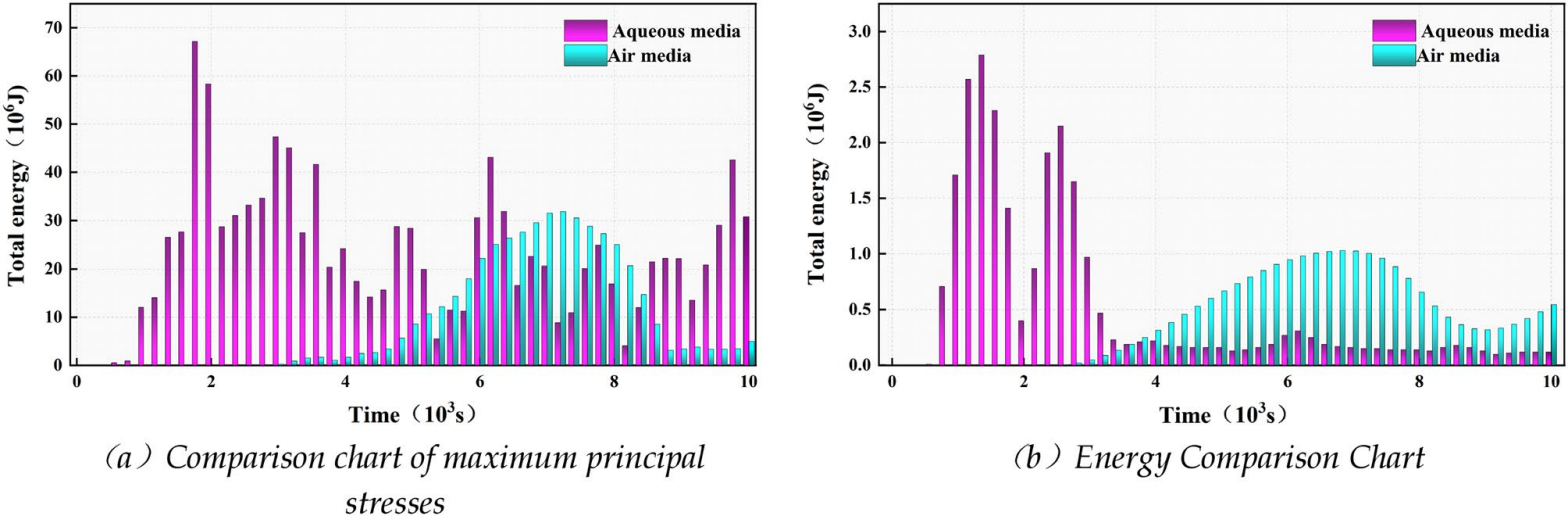
Diagram showing the results of the action of the blasting media.

After the calculations were completed, the central unit of the steel plate was selected to extract the maximum principal stress, and the results in Fig. 10a show that when the blasting medium is water, the maximum principal stress applied to the steel plate exceeds the air medium, with the peak maximum principal stress reaching 1.53 times the peak maximum principal stress of the air medium, and the time of action of the stress also far exceeds that of the air medium and is distributed throughout the process. Figure 10b shows that the peak energy transferred by the water medium can be up to 2.73 times that transferred by the air medium and has a clear advantage at the beginning of the blast. The above proves that the water medium is a good transfer medium and can transfer shock waves and energy well so that the blast object is subject to stronger action and less loss.

### Explosive bubble pulsation phenomena in water

**Figure 11:**
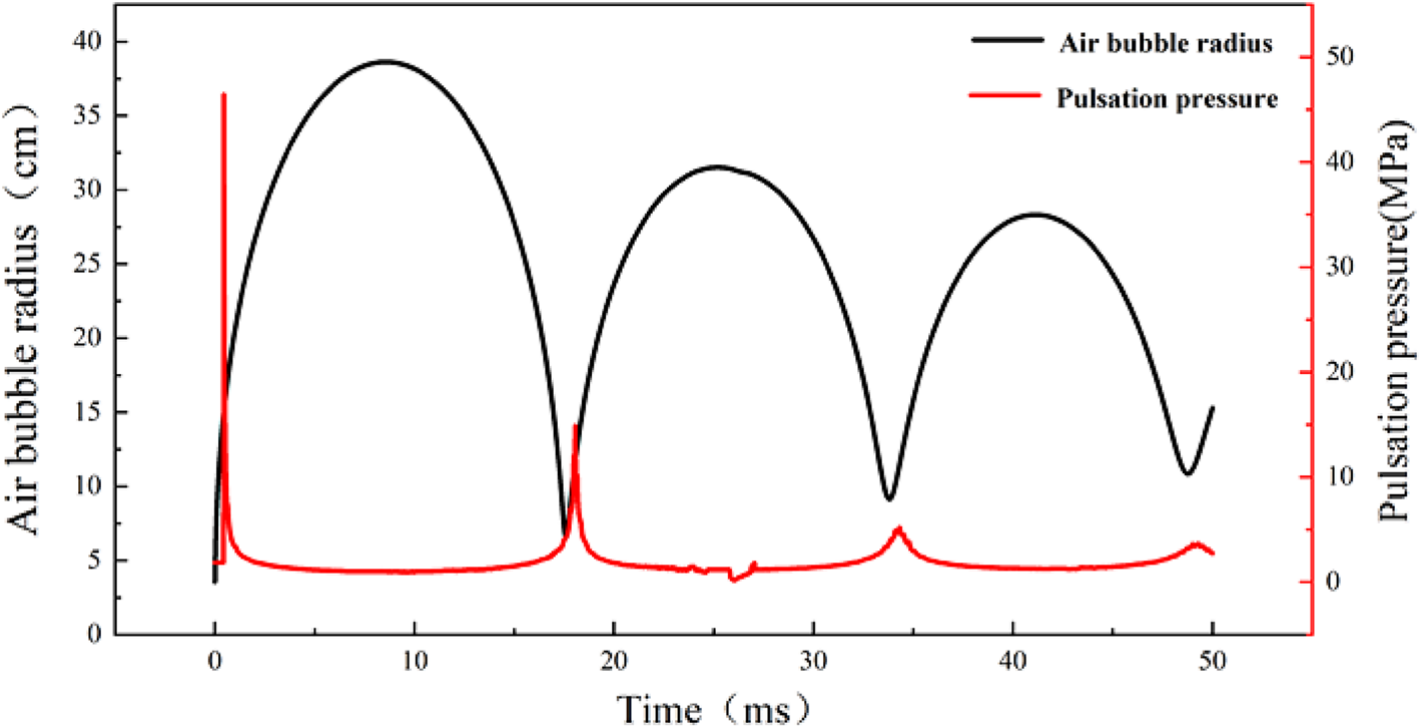
Numerical simulation results of the bubble pulsation phenomenon.

Explosives in the water after the explosion instantly transformed into a rapidly expanding high-temperature, high-pressure air mass, and the formation of shock waves propagating outward in the water, when the bubble expansion due to inertia expansion to the maximum time, the pressure inside the bubble is less than the surrounding hydrostatic pressure to form a negative overpressure, the periphery of the water back to the extrusion of bubbles until compressed to the smallest diameter, at this time, it will be found that the pressure inside the bubble is higher than the external pressure, so that bubbles and start a new round of The expansion and contraction of the cyclic process, so repeated many times to form bubble pulsation phenomenon in Fig. 11. Bubble pulsation radius from 38.64 to 28.58 cm, the bubble pulsation cycle is also due to the reduction of the expansion range from 17.61 to 14.63 ms. such as Fig. 11 shows that the first bubble pulsation peak pressure only reaches the peak pressure of the shockwave of 20% or so, the peak pressure in the water-bearing holes in the blast, there will be explosives in the water to detonate, the explosion of water bubble pulsation will also play a certain role in the destruction.

### Blasting effects of different water contents in natural aqueous media holes

#### Blasting stresses around the perimeter of blasting holes with different water contents in natural aqueous media holes

**Figure 12:**
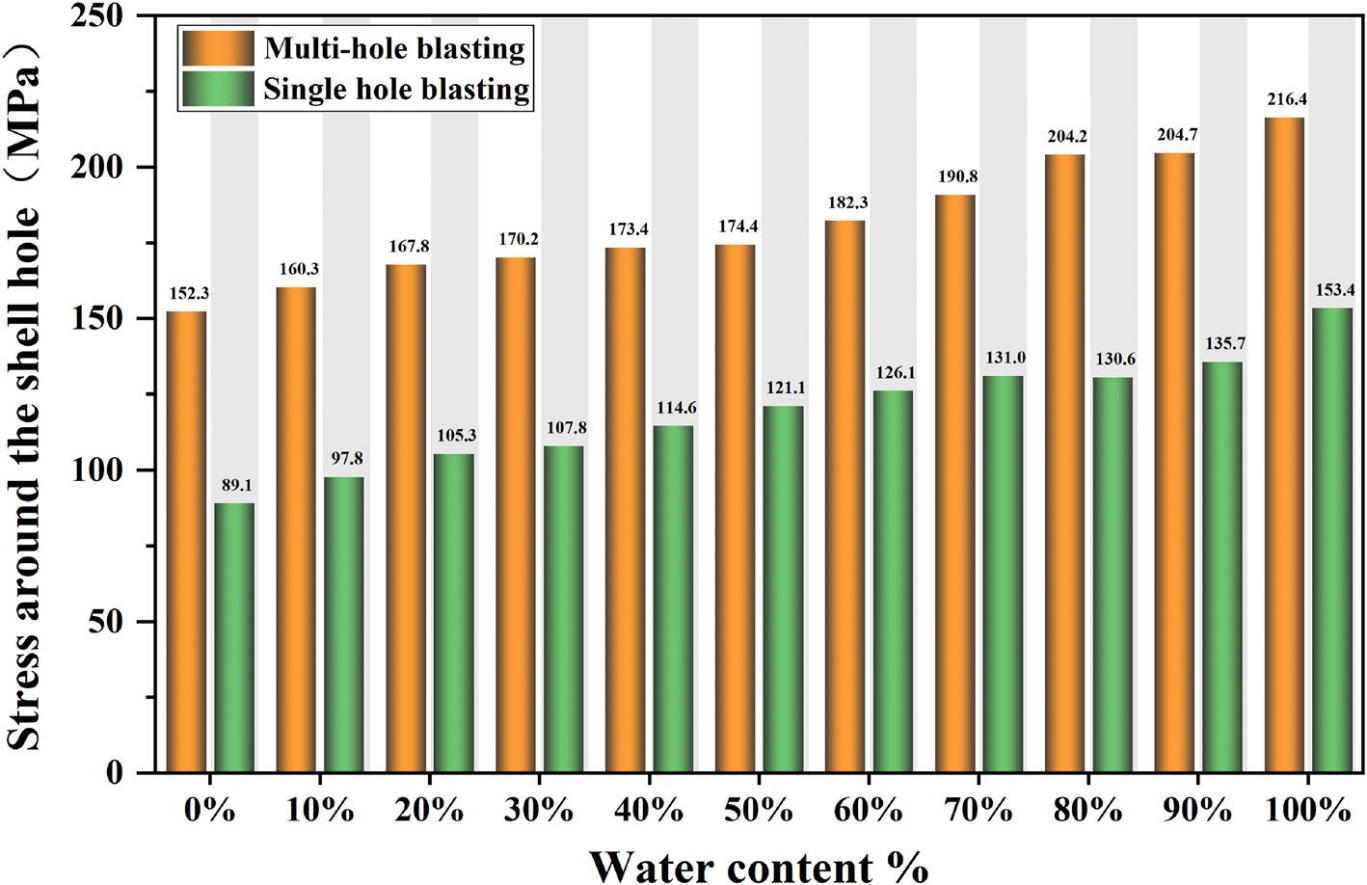
Trend of maximum principal stresses in the perimeter unit of a blast hole containing water.

In order to study the stresses generated by blasting under different water content conditions, the rock mass around the shell hole was selected as the observation object, and the numerical simulation results were extracted by LS-PrePost software for statistical analysis of its maximum principal stresses, and the maximum principal stresses in the perimeter unit of the natural aqueous media hole blasting model are shown in Fig. 12.

In the case of single-hole blasting of natural aqueous media holes, the maximum principal stress in the perimeter unit gradually increases with the increase in water content, with a peak of 89.06 MPa for a 0% charge and 153.42 MPa for a 100% charge, with the maximum peak stress of the latter exceeding that of the former by 72.27%. This means that at the same charge, the greater the water content, the greater the stress generated and the more likely it is to cause damage and destruction to the rock.

In the case of multi-hole blasting of natural aqueous media holes, the change in maximum principal stress in the unit around the gun hole has a similar trend to the change in maximum principal stress in the unit around the gun hole to that of the unit around the single gun hole, with a maximum principal stress of 152.30 MPa at 0% water content and 216.35 MPa at 100% water content. Due to the multiple gun hole blasting when the resulting maximum principal stresses in the multi-hole blasting model increased by an average of 52.14% compared to the single-hole blasting model due to the superimposition of shock waves.

#### Influence of different moisture contents on the forces at the top and foot of the terrace slope

**Figure 13:**
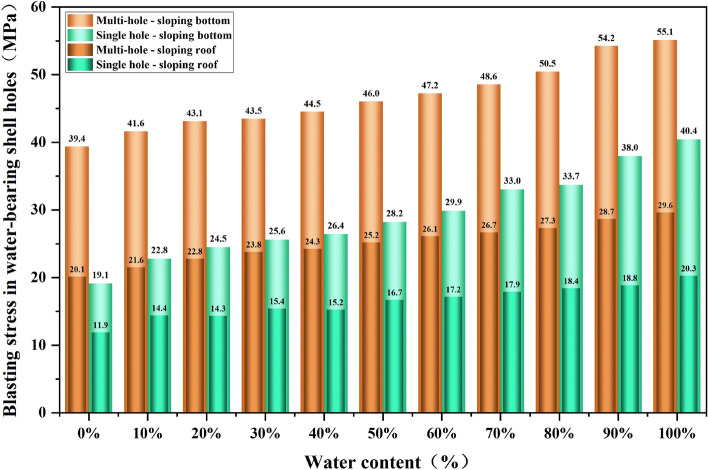
Trend of stresses at the top of the slope toe in gunholes with different water contents.

In order to study the stresses generated by blasting under different water content conditions, the foot of the blasting step and the top of the next step was selected as the observation objects, and the numerical simulation results were extracted by LS-PrePost software for statistical analysis of their maximum principal stresses, and the maximum principal stresses at the top and foot of the slope for the natural aqueous media hole blasting model are shown in Fig. 13.

In the single-hole blasting model for natural aqueous media holes, the maximum principal stresses in the units around the holes had the following characteristics: the maximum principal stresses in the units at the top and foot of the slope increased gradually with the increase in water content, similar to the trend in the units around the holes. The maximum principal stress in the dry hole with 0% water content is the smallest, at 19.14 MPa and 11.90 MPa, while the maximum principal stress in the full water gun hole with 100% water content is the largest, at 40.44 MPa and 20.27 MPa. The maximum principal stress in the perimeter unit decreases significantly. The maximum principal stresses in the screening unit at the foot of the blasting step slope with less than 50% water content failed to exceed the tensile strength of the rock in the model and may not break the rock properly and there is a possibility of leaving a root. In contrast, none of the maximum principal stresses at the top of the lower step slope exceeded the tensile strength of the rock mass, resulting in a low level of damage.

In the natural water-bearing holes in the multi-hole blasting model, the maximum principal stress changes around the gun hole unit has the following characteristics: in the natural water-bearing holes in the multi-hole blasting model, the maximum principal stress at the top of the slope, the foot of the slope unit compared to the single-hole blasting model more than double the maximum principal stress of the bottom of the slope unit is also more than the tensile strength of the rock mass, so that the original possible root problem can be solved, from Fig. 13 also know that the water media blasting in the foot of the slope is greater than the top of the slope.

### Field test results

**Figure 14:**
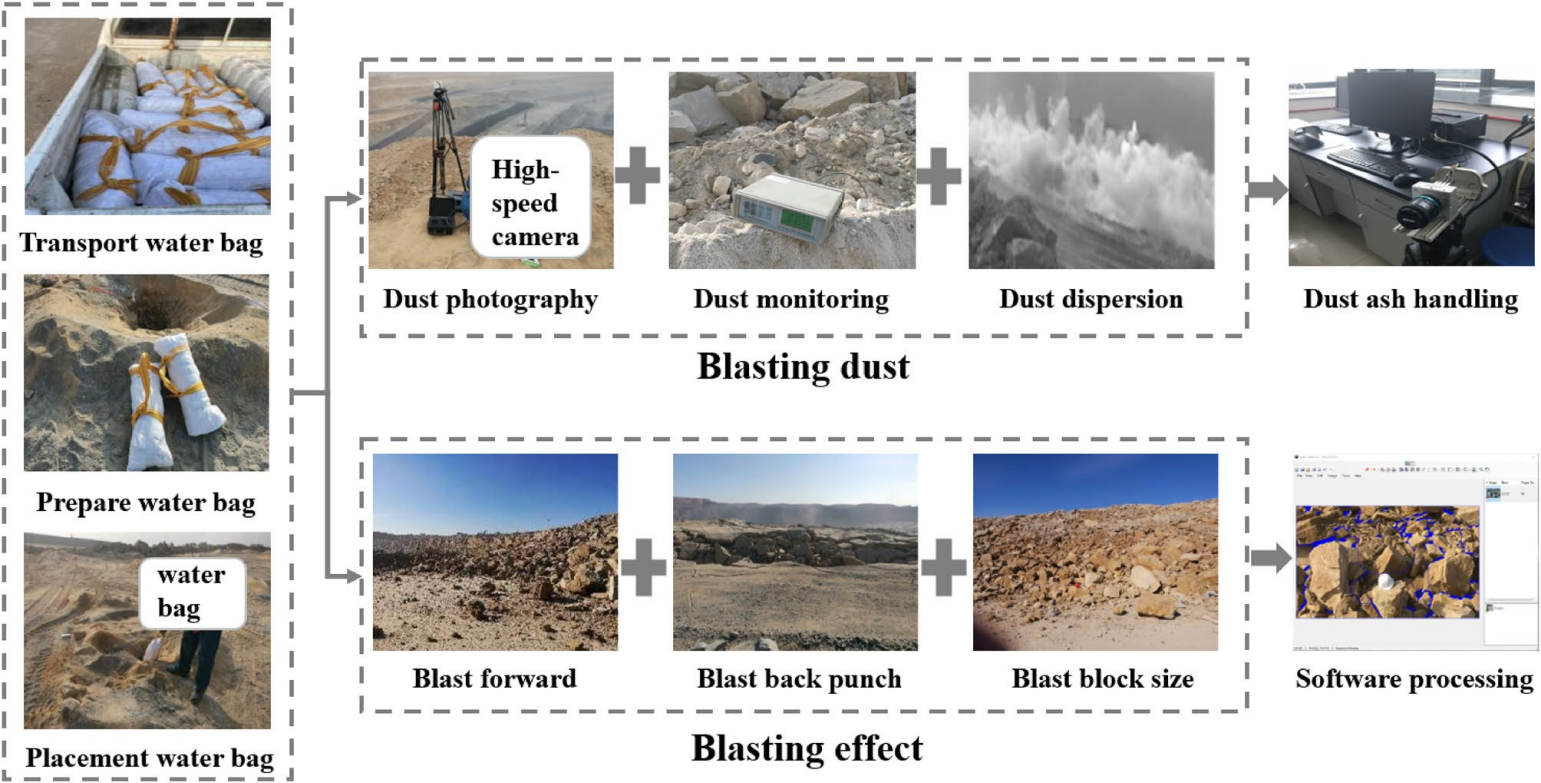
Effect of aqueous media blasting on site.

The field test was carried out at the edge of the blast area for the filling of the aqueous media, as shown in Fig. 14 before the test, the water bags were transported from the warehouse to the blast area for the filling of water bags, the bags were filled and processed for protection, and suitable points were selected to be photographed directly to the blast area using a high speed camera to prepare for the recording of the whole blasting process, and at the same time a dust concentration monitor was placed 100 m downwind from the blast area edge at the blast point The dust concentration was measured by a dust monitor and the dust images were processed using the MATLAB software grey scale recognition function; after the blast was completed, the blasting area was entered and photographed to obtain post-blast step coordinates and blast effect images for blast effect analysis using Split-Desktop open pit blast block analysis software.

#### Effectiveness of blasting dust reduction in shell holes containing aqueous media

**Figure 15:**
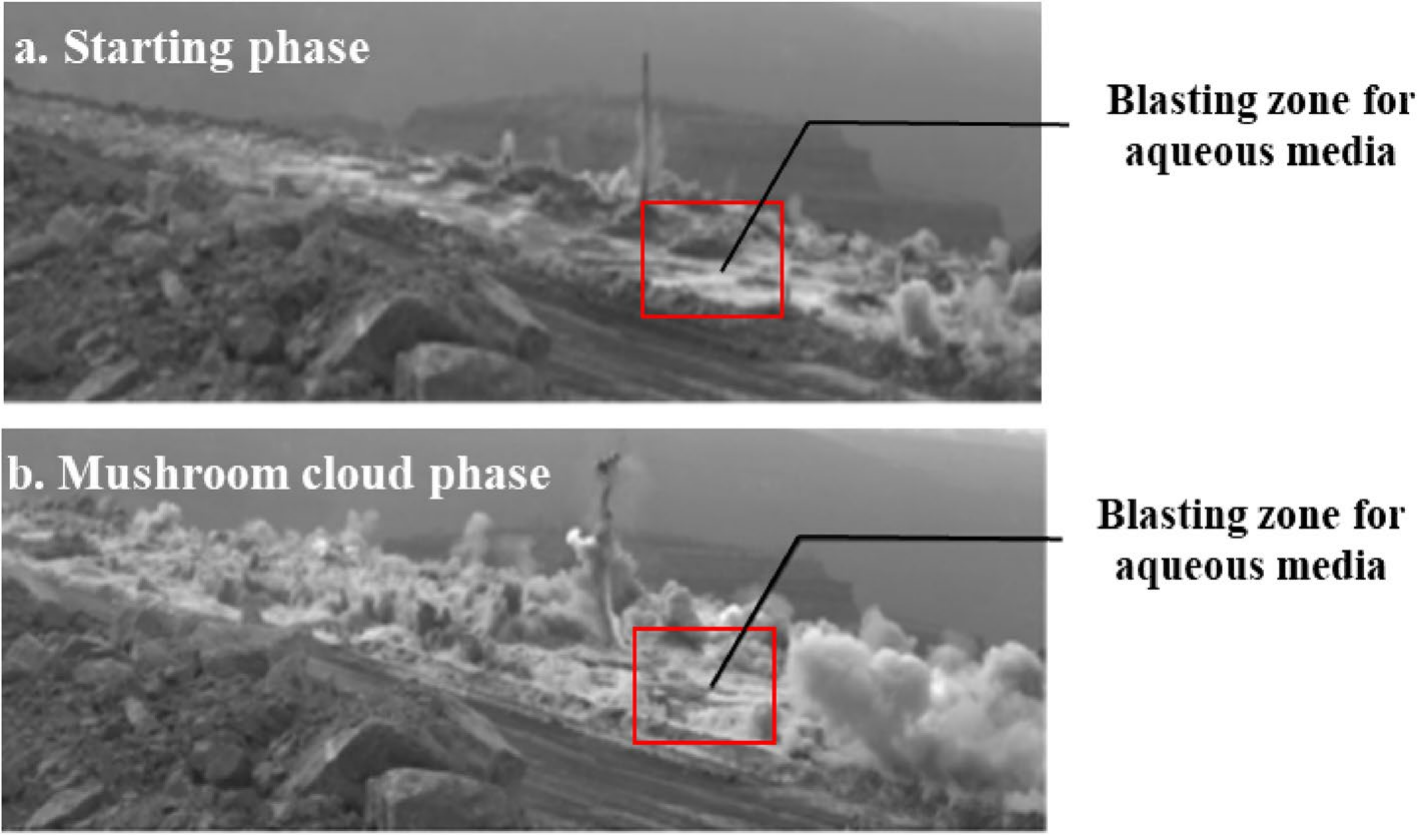
Effect of aqueous media blasting.

As shown in Fig. 15 from the high-speed camera, the dust reduction effect of this test is obvious, with the amount of dust in the area of the water-filled borehole being less than that in the area of the unfilled borehole. During blasting, the water bag in the hole was broken by the blast wave and part of the water solution was spread evenly around the hole, isolating and adsorbing the dust in the jumping phase. The amount of dust generated in the aqueous media blasting area during the jump and mushroom cloud phases of the blast was minimal, in contrast to the blasting area where the water bags were not filled. As a result of the experimental design concept and the actual deployment process to avoid the influence of external disturbing factors, the effect of this aqueous media blasting on dust reduction is ideal and the blasting solution is effective in the blasting of rock in opencast mines.

**Figure 16:**
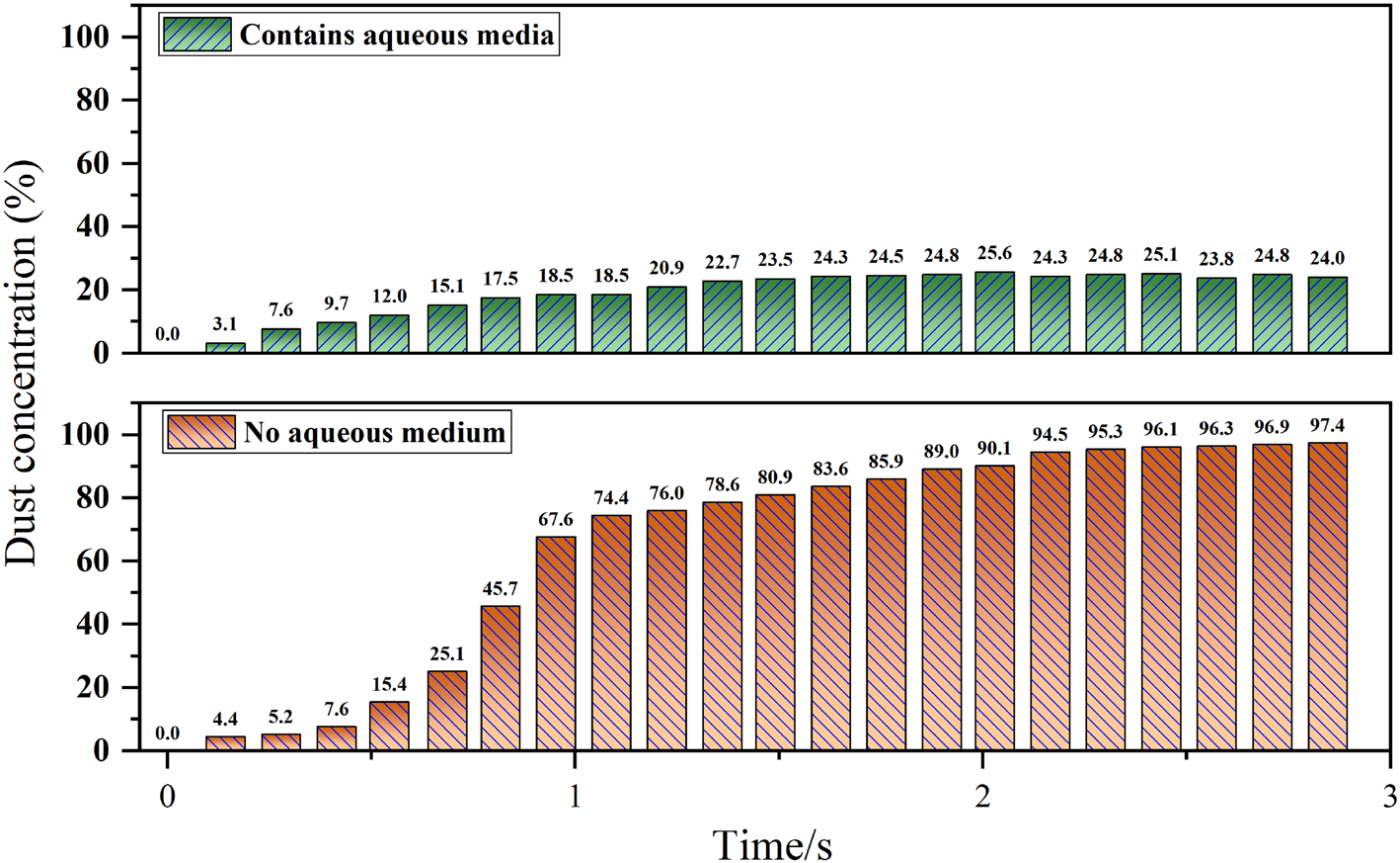
Comparison of changes in dust concentration.

In order to obtain detailed data on the effect of aqueous media blasting on dust reduction, MATLAB software was used to count the distribution of the grey value of the selected blasting pictures, intercept the pictures of the blasting test area with and without aqueous media location and carry out the grey value statistics respectively to characterise the dust concentration as a percentage of the grey value at different times, with time as the horizontal axis and dust concentration as the vertical axis, the statistical results of all pictures were plotted in the coordinate system to represent the change characteristics of dust concentration with time, as shown in Fig. 16.

Analyzed from Fig. 16 can be concluded that the water-mediated area and non-aqueous media area dust concentration have experienced the three stages of blasting dust, but there is a water-mediated area of the dust concentration change is relatively gentle. Overall, the water media than non-aqueous media zone dust concentration is much lower, it can be inferred that due to the blast effect of water bag rupture, water in the water bag is spread into the air and dust particles fully combined. Calculate the average of the dust concentration in the two states, we can get the effective dust reduction rate of 75% of the water-mediated dust reduction program.

#### Comprehensive evaluation of the blasting effect of shell holes containing aqueous media

After using special mining measuring equipment to measure the nine factors affecting blasting data as shown in Table 9, integrated shoveling, crushing and other on-site construction requirements, the critical size of the test bench block was positioned at 1.2 m. After blasting tests were carried out on-site, data collection, blast pile photos were taken, followed by blast pile photos using Split-desktop block analysis software processing and analysis.

**Table 9:** Statistics on factors affecting blasting effectiveness in blasting tests.

Test number	Chunk rate /%	Pre-impulse volume /m	Backlash /m	Backwash slit /m	Root rate /%	Explosives unit consumption /(kg/m^3^)	Other consumables cost per hole/¥	Single hole loading time /min	Shovelling efficiency /(m^3^/h)
Original blast 1	3.35	22.3	3.51	1.4	6.51	0.49	45.63	12.15	517.39
Original blast 2	3.25	23.6	3.2	1.2	6.42	0.47	44.95	11.67	492.98
Aqueous media blasting 1	2.83	19.4	1.97	0.8	6.21	0.43	53.83	15.67	512.65
Aqueous media blasting 2	2.77	20.7	2.21	1.1	6.19	0.41	50.88	14.87	520.33
Aqueous media blasting 3	2.89	19.3	1.86	0.8	5.97	0.45	52.12	16.31	528.18

Based on the actual demand for test open-air step blasting and historical blasting work records, a summary analysis was carried out to derive a grading scale for each evaluation indicator and establish an indicator grading scale Table 10.

**Table 10:** Grading scale for evaluation indicators.

Factors	v_1_	v_2_	v_3_	v_4_	v_5_	Weighting
	90–100	75–90	60–75	45–60	0–45	
Chunk rate (%)	≤ 3%	3%–6%	6%–9%	9%–12%	≥ 12%	0.212
Pre-impulse volume (m)	≤ 10	10–20	20–30	30–40	≥ 40	0.090
Backlash (m)	≤ 3	3–6	6–9	9–12	≥ 12	0.090
Backwash Slit (m)	≤ 1	1–2	2–3	3–4	≥ 4	0.079
Root rate (%)	≤ 5%	5%–10%	10%–15%	15%–20%	≥ 25%	0.068
Explosives unit consumption (kg/m^3^)	≤ 0.3	0.3–0.6	0.6–0.9	0.9–1.2	≥ 1.2	0.223
Other consumables (cost per hole/¥)	≤ 50	50–75	75–100	100–125	≥ 125	0.074
Construction efficiency (single hole filling time/min)	≤ 10	10–15	15–20	20–25	≥ 25	0.041
Shoveling efficiency (m^3^/h)	≥ 550	500–550	450–500	400–450	≤ 400	0.123

**Figure 17:**
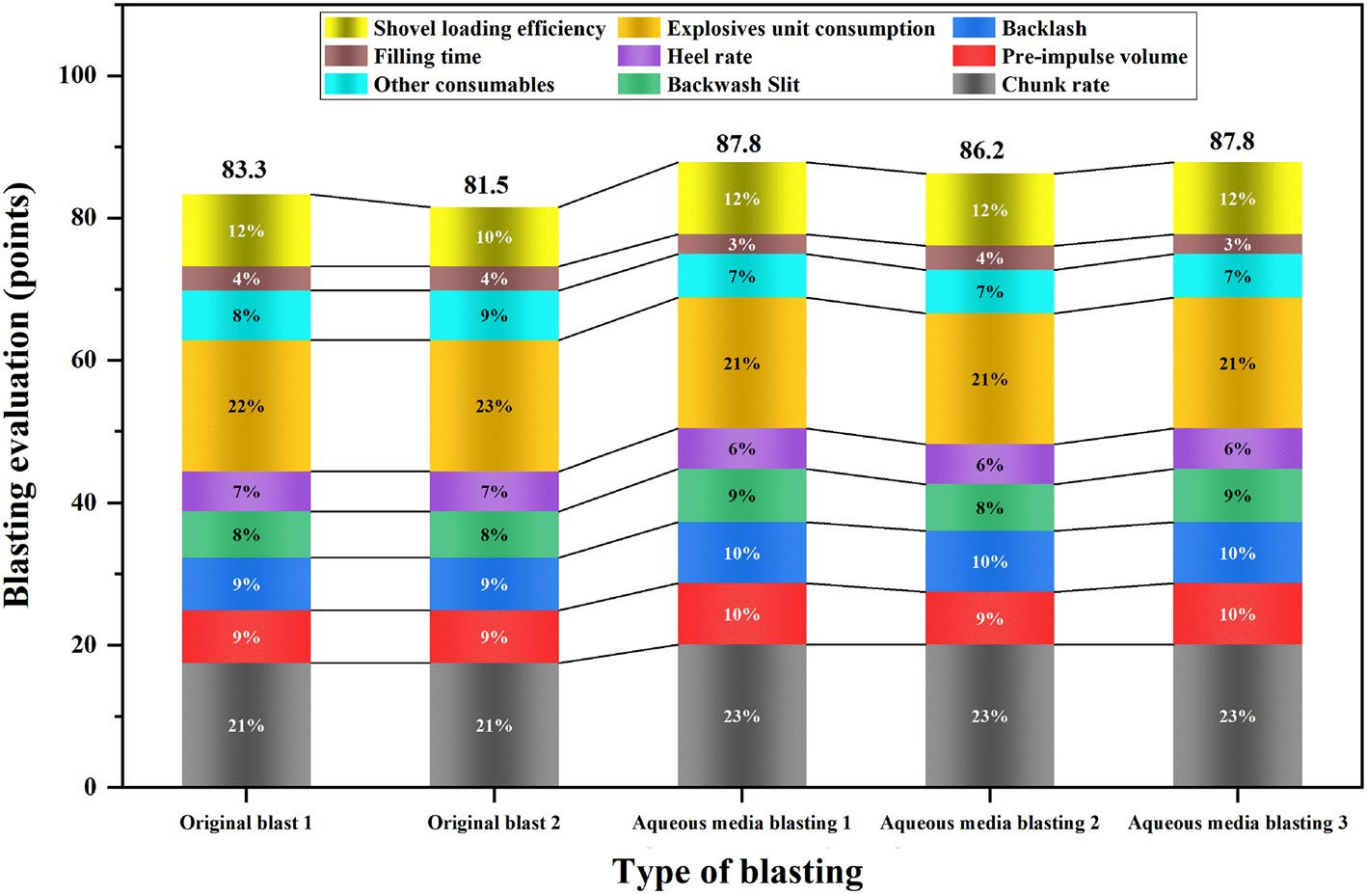
Combined blast test score statistics.

Using the above data information to build up the evaluation matrix for each trial for evaluation, combined with the evaluation index weights obtained by hierarchical analysis to obtain evaluation vectors, according to the principle of maximum affiliation to obtain the final evaluation and quantify it in detail to obtain the following evaluation, see Fig. 17 for details.

In summary, the five blasts described above were all good blasts, with the final blast effect scoring the aqueous media blast test to exceed the original blast design due to the high weighting given to bulk rate, explosive unit consumption, and shovel loading efficiency. It can be seen that aqueous media blasting has practical engineering value.

## Conclusions

In this paper, from the mechanical characteristics of water-mediated blasting combined with water-mediated blasting site effects, water-mediated blasting of dry holes in the mine site, verified that it is an excellent charging program, in the control of block, save blasting costs have outstanding performance, can well enhance the quality of blasting, has good blasting effects, in the actual project can be considered without dealing with the water medium in the rock body directly water medium uncoupled charging. After researching this paper, we have obtained the following conclusions:

1. this paper analyzes the water medium in the blasting process transfer role, energy transfer role, bubble pulsation phenomenon, the results show that the blasting medium for the water medium, the maximum principal stress for the air medium 1.53 times; transfer of energy peak can be up to 2.73 times that of the air medium.
2. With TrueGrid/LS-DYNA software to simulate the dynamic process of blasting, the study of hole perimeter, top of the slope, foot of the maximum principal stress changes, the results show that in the natural water holes in the hole perimeter, top of the slope, foot of the maximum principal stress of the unit with the increase in water content is gradually increasing trend.
3. the water-mediated blasting field test using image analysis, comparing the water-mediated blasting area and the water-free media blasting area pictures of the gray value, through the error correction, the water-mediated blasting area and the water-free media blasting area of the dust concentration compared to the curve of the change, after calculating the water-mediated blasting test program is about 75% of the rate of dust reduction.
4. The use of AHP-fuzzy comprehensive evaluation method of two groups of traditional dry hole blasting and three groups of water-mediated blasting comprehensive evaluation, the results show that the water-mediated blasting scores are higher than the traditional dry hole blasting, proving that water-mediated blasting has a certain engineering application prospects.

## Data Availability

The datasets generated and/or analysed during the current study are not publicly available due [The data supporting the conclusions of this study were obtained from the mines, which are restricted in their availability, and, permission was obtained for their use in this study. Mine data are confidential and not available to the public, and therefore not available.] but are available from the corresponding author on reasonable request.
